# Metabolic Regulation of T cell Activity: Implications for Metabolic-Based T-cell Therapies for Cancer

**DOI:** 10.52547/ibj.3811

**Published:** 2022-12-19

**Authors:** Jahangir Abdesheikhi, Farnaz Sedghy, Merat Mahmoodi, Hossein Fallah, Mahdi Ranjkesh

**Affiliations:** 1 Department of Immunology, School of Medicine, Kerman University of Medical Sciences, Kerman, Iran;; 2 Department of Biochemistry, School of Medicine, Kerman University of Medical Sciences, Kerman, Iran

**Keywords:** Cancer, metabolic pathways, metabolism, T-cell

## Abstract

Immunometabolism is an emerging field in tumor immunotherapy. Understanding the metabolic competition for access to the limited nutrients between tumor cells and immune cells can reveal the complexity of the tumor microenvironment and help develop new therapeutic approaches for cancer. Recent studies have focused on modifying the function of immune cells by manipulating their metabolic pathways. Besides, identifying metabolic events, which affect the function of immune cells leads to new therapeutic opportunities for treatment of inflammatory diseases and immune-related conditions. According to the literature, metabolic pathway such as glycolysis, TCA cycle, and fatty acid metabolism, significantly influence the survival, proliferation, activation, and function of immune cells and thus regulate immune responses. In this paper, we reviewed the role of metabolic processes and major signaling pathways involving in T-cell regulation and T-cell responses against tumor cells. Moreover, we summarized the new therapeutics suggested to enhance anti-tumor activity of T cells through manipulating metabolic pathways.

## INTRODUCTION

Immunometabolism, a term first used in 2011, is a new field of research that seeks to improve our understanding of the multifaceted relationship between the metabolic and the immune system, from metabolic features of the immune cells to metabolic disorders by these immune cells^[^^1^^,^^2^^]^. Over the past decade, immunologists have focused on altering metabolic pathways within immune cells to enhance their optimal function^[^^3^^]^. Also, identifying metabolic events affecting the function of immune cells provides new therapeutic opportunities for treatment of the immune-related and inflammatory diseases^[^^4^^]^. Metabolic processes, such as glycolysis, TCA cycle, and fatty acid metabolism, notably influence the function of immune cells and are now considered as the main factors regulating the immune responses^[^^4^^]^. This review aims to understand the role of metabolic pathways in T-cell responses, especially against tumor cells; we also provide the present knowledge on how the antitumor activity of T cells can be enhanced through manipulating the metabolic events.

**Six major metabolic pathways**

**Fig. 1:**
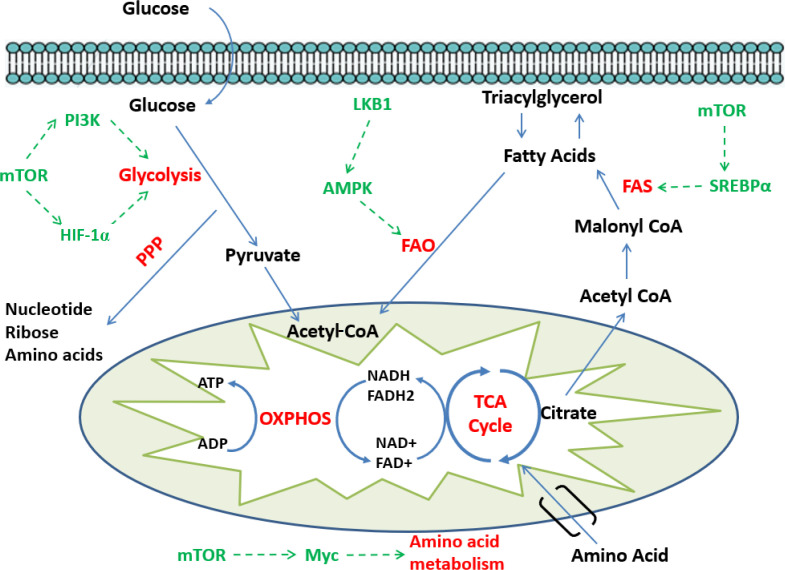
Major metabolic and signaling pathways in immunometabolism. During the glycolysis pathway, glucose is converted to pyruvate, which can trigger the TCA cycle. NADH and FADH2 produced in this cycle deliver their electrons to the electron transport chain, which finally leads to ATP production. The pentose phosphate pathway (PPP) converts glycolytic pathway intermediates to compounds such as ribose to produce nucleotides, amino acids, and NADPH. Citrate removed from the TCA cycle is used together with NADPH for fatty acid synthesis. Fatty acids can also be oxidized to produce NADH and FADH2, which again leads to the production of ATP from the electron transport chain. Finally, amino acid metabolism provides energy for cell growth and protein biosynthesis through the TCA cycle. Under nutrient deficiency conditions, LKB1-AMPK pathway signaling inhibits glycolysis and FAS pathways as well as increases fatty acid oxidation. During the activation or in nutrient-rich conditions, PI3K-Akt and mTORC1 signaling increases glycolysis, fatty acid synthesis, and amino acid metabolism. SREBP-α, sterol regulatory element-binding protein-α; ADP, adenosine diphosphate

The major metabolic pathways involved in the survival, proliferation, and activation of the immune cells contain glycolysis, pentose phosphate pathway, TCA cycle, fatty acid oxidation, fatty acid synthesis, and amino acid synthesis, particularly tryptophan, arginine, and glutamine^[^^5^^]^ (Fig. 1). 

***Glycolytic metabolic pathway***

In glycolytic metabolism, two molecules of pyruvate are produced from each molecule of glucose, resulting in only two molecules of ATP. It is considered a relatively inefficient pathway for the production of cellular ATP. However, the glycolytic pathway does not require oxygen and can occur in both aerobic and anaerobic conditions^[^^6^^,^^7^^]^. Furthermore, it provides other imperative factors for cell survival by promoting the production of NADH, a cofactor of various enzymes, and shifting some byproducts to other biosynthetic pathways to support anabolic growth, including glucose-6-phosphate for the synthesis of ribose during the pentose phosphate pathway, 3-phosphoglycerate for the production of amino acids during the biosynthesis of serine, and pyruvate for the synthesis of citrate in the Krebs cycle^[^^6^^]^.

***Pentose phosphate pathway***

The pentose phosphate pathway shifts the mediators of the glycolytic pathway to the production of nucleotide and amino acid precursors that are necessary for cell growth and proliferation^[^^8^^]^. This pathway involves the oxidation of glucose but is an anabolic rather than a catabolic pathway, generating NADPH, pentose, and ribose-5-phosphate^[^^5^^]^. Pentose is produced in the non-oxidative phase, while NADPH is generated in the oxidative phase and used to produce reactive oxygen species during respiratory explosion as well as glutathione and other antioxidants^[^^5^^]^.

***Tricarboxylic acid cycle pathway***

The TCA cycle (citric acid cycle or Krebs cycle) is a metabolic pathway appropriate for supplying cellular ATP and CO_2_ through oxidation of acetyl-CoA, derived from carbohydrates, fatty acids, and proteins^[^^9^^]^. The TCA cycle and OXPHOS are the principal sources for ATP production in cells and need more energy and longevity^[^^6^^]^. Other byproducts of the TCA cycle, including NADH and FADH2, support OXPHOS and highly efficient for ATP production by transferring electrons to oxygen during cellular respiration; moreover, citrate generates N-acetylglucosamine uridine diphosphate and succinate, two essential substances for the physiological function of immune cells^[^^9^^]^.

***Fatty acid oxidation pathway***

The FAO pathway converts fatty acids into several products, including acetyl-CoA, NADH, and FADH2, that are needed to produce cellular energy^[^^6^^]^. Acetyl-CoA then enters the TCA cycle, while NADH and FADH2 transfer electron to the coenzyme Q of the electron transport chain, which eventually produces ATP^[^^10^^]^.

***Fatty acid synthesis pathway***

The FAS pathway leads to production of needful lipids for cell growth and proliferation^[^^6^^]^. This pathway uses the products of other metabolic pathways such as glycolysis, TCA cycle, and pentose phosphate pathway^[^^6^^]^. The major substrate for synthesis of fatty acids is cytoplasmic acetyl-CoA that is converted to malonyl-CoA. Seven molecules of malonyl-CoA are then combined with an acetyl-CoA via the fatty acid synthase enzyme to form palmitate, which finally produces other types of fatty acids^[^^11^^]^.

***Metabolic pathways of amino acids***

Amino acid metabolism plays an important role in different aspects of cell biology^[^^6^^]^. Glutamine, arginine, and tryptophan are the key amino acids studied in the immune system. For instance, glutamine and aspartate are required for the synthesis of purine and pyrimidine. Glutamine may also be utilized by active proliferating cells as an alternative to entering the TCA cycle, where it involves in ATP production or fatty acid synthesis. Other amino acids, including arginine and tryptophan, are metabolized through several other pathways to support cell proliferation and anabolic processes^[^^6^^,^^12^^]^. Taken together, all the mentioned metabolic pathways possess unique purposes within the cell and are regulated by specific signaling pathways, depending on the cell requirements.

**Major signaling pathways in immunometabolism**

Major signaling pathways involve in immunometabolism are PI3K/AKT, mTOR, and LKB1-AMPK^[^^13^^]^.

***PI3K-Akt signaling in T-cell activation***

Following T-cell activation, signaling mediated by TCR, CD28, and IL-2R leads to the phosphorylation and activation of PI3K, as well as inactivation of PI3K suppressor molecules such as PTEN and phosphoinositide-3-kinase interacting protein^[^^13^^]^. PI3K converts PIP2 to PIP3, a factor that facilitates the activation of downstream molecules, including Akt. Finally, the activation of Akt increases the metabolism and the activation of T cells^[^^13^^,^^14^^]^.

***mTOR signaling in T-cell activation***

mTOR is a member of the phosphatidylinositol 3-kinase family of kinases, and the main component of two distinct protein complexes, including mTORC1 and mTORC2. These complexes regulate diverse cellular processes such as growth, proliferation, stimulation, survival, autophagy, and transcription^[^^13^^]^. mTORC1 is sensitive to nutrients such as amino acids, and upon activation, it promotes cell growth, protein transport, lipid synthesis, and autophagy inhibition. On the other hand, mTORC2 acts as a protein tyrosine kinase that activates insulin receptors and insulin-like growth factor receptors. It is also involved in the organization of the cytoskeleton^[^^15^^]^.

***LKB1-AMPK signaling pathway in T cell activation***

LKB1 is a serine/threonine kinase that acts as a tumor suppressor and inhibits the proliferation and metabolism of cancer cells through phosphorylating and activating AMPK^[^^16^^]^. The LKB1-AMPK signaling pathway plays a substantial role in regulating cellular metabolism, proliferation, and survival in response to nutrients^[^^17^^]^. The signaling of AMPK inhibits metabolic pathways such as glycolysis^[^^18^^]^, glutaminolysis^[^^13^^]^, and lipogenesis^[^^19^^]^ while enhances catabolic processes like mitophagy and autophagy^[^^13^^]^. 

***Cross-regulation of signaling pathways***

The signaling pathways can regulate each other; for instance, in nutrient-rich conditions, activating the PI3K-Akt and mTORC1 signaling pathways increases glycolysis, mitochondrial development, and fatty acid synthesis, but suppresses autophagy. Akt activation leads to the phosphorylation of LKB1 and suppression of its function. This regulation has not yet been reported in T cells^[^^20^^]^. Under a nutrient-deficient condition, LKB1-AMPK signaling inhibits glycolysis

and fatty acid synthesis while elevates mitochondrial homeostasis and autophagy. However, the modulation of these mechanisms in T cells needs further investigation^[^^13^^]^. Understanding the signaling pathways regulating immunometabolism may help discover therapeutic strategies to target metabolic mediators and enhance the T-cells responses in human diseases, including cancer (Fig. 1). 

**Metabolic regulation during T cell evolution**

During the development of T lymphocytes in the thymus, the proliferation of double-negative thymocytes, along with the activation of the PI3K-AKT signaling transduction, increases the expression of glucose transporter, Glut1, which promotes glycolysis pathway. Therefore, double-negative thymocytes transit to double-positive thymocytes^[^^21^^,^^22^^]^. At this stage, the expression of Glut1, and subsequently the proliferation rate and metabolic activity of double-positive thymocytes decreases^[^^23^^]^. Finally, during the transition to the single positive T cells, glycolysis reduces, and shifts to FAO and OXPHOS pathways^[^^23^^,^^24^^]^.

**Metabolic regulation of naive T cells**

**Fig. 2:**
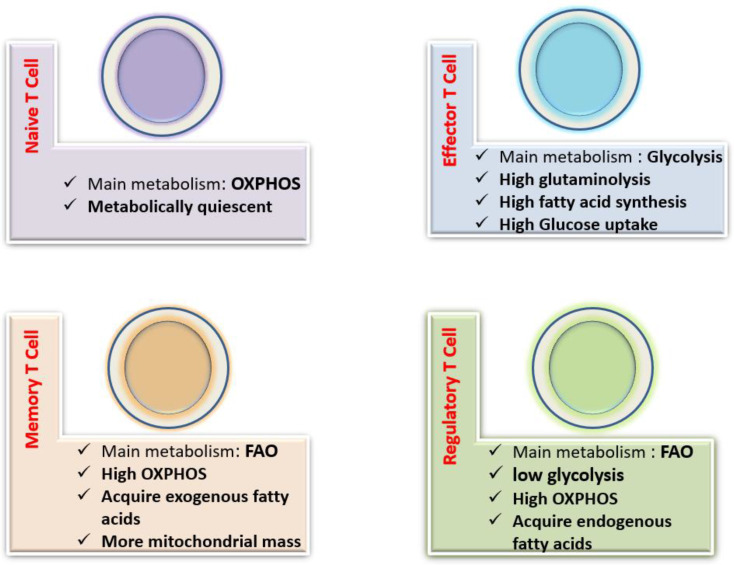
Metabolic pathways of T cell subtypes. Different T cell subsets rely on distinct metabolic pathways to promote cell survival, lineage generation, and function. Naïve T cells mainly use the OXPHOS metabolic pathway. Effector T cells use glycolysis, fatty acid synthesis, and glutaminase to proliferate and produce cytokine. Treg cells utilize OXPHOS and fatty acid oxidation. Similarly, memory T cells also require OXPHOS and FAO to increase cell lifespan

The primary function of naive T cells is antigen monitoring, which requires a relatively small amount of ATP to support processes such as ion homeostasis, membrane adhesion, and actin cytoskeleton rearrangement. Thus, naive T cells bear a rather catabolic profile and tend to produce energy through OXPHOS^[^^25^^]^. In addition to glucose, glutamine and fatty acids are considered the main sources of fuel for naive T cells. Under glucose deficiency, glutamine could be a source of ATP to facilitate naive T-cell proliferation^[^^26^^]^ (Fig. 2). 

**Metabolic regulation of effector T cells**

Effector T cells prefer glycolysis and OXPHOS metabolism, indicating higher metabolic needs than naive T cells^[^^25^^]^. Unlike naive T cells, high expression of Glut1 is of special importance in the survival of effector T cells. However, evidence has shown that these cells are not completely dependent on glucose to produce ATP. A study in mice has reported that effector T cells use glutamine to maintain intracellular ATP levels in glucose-deficient conditions^[^^27^^]^, but another study has highlighted the role of Glut1 expression and glycolysis in the differentiation of effector T cells^[^^28^^]^. While both CD8^+^ T and CD4^+^ T subsets augment glycolysis and glucose transporter Glut1 expression, CD8^+^ T cells perform less glycolytic activities than CD4^+^ T cells and, thus, rely more on the OXPHOS for their effector function^[^^29^^]^ (Fig. 2). 

***Glucose metabolism in effector T cells***

The mTOR is considered as the major regulator of effector T cell metabolism by modulating glycolysis. When T cell is activated, triggering the PI3K/Akt signaling pathway can augment mTOR signaling and rises the expression of HIF-1α, the key player in response of cells to hypoxic conditions. HIF-1α impresses different downstream targets including nutrient receptors (such as GLUT-1, SLC1a5, and Solute Carrier Family 1 Member 5) to enable cells to uptake and utilize glucose during glycolysis^[^^30^^]^. According to the available data, this pathway predominantly induces the differentiation to Th17 cells through direct interaction with the IL-17 promoter^[^^31^^]^; indeed, mTORC1 predominantly regulates the differentiation into Th1 and Th17, and mTORC2 is involved in differentiation into Th2 subset^[^^15^^]^. Other factors activated by mTOR is Myc transcription factors, present in the downstream of TCR signaling to regulate glycolysis and glutaminolysis in T cells^[^^32^^]^. In addition to mTOR, the IRF4 controls the glycolytic pathway of effector T cells, as in the absence of IRF4, glucose uptake and glycolysis are impaired in the activated CD4^+^ T cells^[^^33^^]^. Glycolysis can control the production of IFN-γ from T cells by the glycolytic enzyme GAPDH. This enzyme inhibits the translation of IFN-γ by binding to the 3′UTR region in the related mRNA^[^^27^^]^. Also, LDHA regulates the production of IFN-γ in a 3′UTR-independent mechanism. Increased conversion of pyruvate to lactate leads to less dependence of the cell on OXPHOS and the TCA cycle for energy production. Afterwards, transferring of the citrate from mitochondria to the cytosol and its conversion to acetyl-CoA in the cytosol results in histone acetylation and transcription of the IFN-γ gene^[^^34^^]^. Overall, these studies highlight the vital role of metabolic features in determining the phenotype of the cell.

***Amino acid metabolism in effector T cells***

Amino acid metabolism can affect the activity and the differentiation of T cells. For example, the high levels of glutamine and its metabolite, alpha-ketoglutarate, stimulate the mTORC1 signaling pathway to differentiate into Th1 and Th17 subsets^[^^35^^,^^36^^]^. In contrast, diminished glutamine and leucine metabolism leads to a decrease in mTORC1 activity and cellular Myc expression, thereby, blocking T-cell activation and differentiation into Th1 and Th17, while maintaining Treg cells^[^^37^^,^^38^^]^. According to the literature, effector CD4^+^ T cells, including Th1, Th2, and Th17, rely mainly on glycolysis and glutaminolysis, while Treg cells use lipid oxidation pathway^[^^39^^]^. Together, these studies suggest that the uptake and utilization of amino acids are required for proliferation and optimal performance of the effector T cells.

***Fatty acid metabolism in effector T cells***

Effector T cells use several anabolic pathways to achieve appropriate cellular responses. For instance, high glucose catabolism leads to the production of short chains of carbon molecules that can be transferred to the TCA cycle and increases FAS. In this context, Berod et al.^[^^40^^] ^have observed that blocking FAS rate-limiting enzyme ACC1, disrupts Th17 differentiation in both mouse and human T cells; in addition, knock-out of the ACC1 gene in murine T cells, protects animals against Th17-mediated autoimmune encephalomyelitis 

**Metabolic regulation of memory T cells**

During an inflammatory response, effector T cells need high metabolic activity for their proliferation and function. At the peak of a response, a metabolic shift from anaerobic glycolysis in effector T cells to FAO is required to generate a memory T cell population^[^^21^^,^^41^^]^. Memory T cells primarily use FAO oxidation as their main energy source, which is regulated by tumor necrosis factor 6 receptor and IL-15. TRAF6 increases FAO by activation of adenosine monophosphate-activated kinase^[^^21^^,^^42^^]^, while IL-15 does the same by up-regulating the FAO rate-limiting enzyme, CPT1, and inducing mitochondrial biogenesis^[^^43^^]^. Finally, we can conclude that changes in metabolism and mitochondrial dynamics are critical for the development, longevity, and secondary response in memory T cells (Fig. 2). 

**Metabolic regulation of regulatory T cells**

The differentiation into Treg cells is highly dependent on the mitochondrial FAO metabolism, and a high rate of glycolysis prevents their generation. Treg cells also rely on OXPHOS for their survival and bear more mitochondrial mass and reactive oxygen species production than effector T cells^[^^28^^,^^44^^]^. In comparison with memory T cells, Treg cells primarily prefer exogenous fatty acids for FAO and need lower FAS metabolism^[^^40^^]^. Indeed, both Treg and memory T cells perform FAO as a basal metabolism, but Treg cells also require some aerobic glycolysis to boost their suppressive function^[^^45^^]^. Activation of AMPK results in more Treg differentiation both *in vivo* and *in vitro* by increasing FAO levels^[^^28^^]^. FOXP3 is the main transcription factor of Treg cells that limits glycolysis by blocking the signaling of mTOR, expression of Glut1, and glycolytic enzymes, but promotes FAO by rising the expression of the CPT1 enzyme^[^^46^^]^. PTEN is an intracellular phosphatase that supports Treg function and prevents autoimmunity by stabilizing FOXP3 expression and inhibiting mTOR signaling, and as a result, hindering glycolysis. Deletion of PTEN gene from Treg cells of mice leads to dysfunction of Tregs and consequently, systemic autoimmunity, and lymphoproliferative disorders^[^^47^^]^. Similarly, protein phosphatase 2 is another intracellular phosphatase that enhances Treg function by inhibiting mTOR and glycolysis^[^^48^^]^ (Fig. 2). 

**Effects of tumor metabolism on T cells**

Tumor metabolism refers to changes in the metabolism pathways of tumor cells compared to most normal cells. Tumor cells mainly use glycolysis to produce energy, which causes acidification and lacking the oxygen in the tumor microenvironment^[^^30^^,^^49^^]^. These highly proliferating cells compete with T cells to uptake extra amounts of urgent nutrients, including glucose, amino acids, and fatty acids , which leads to food deprivation, hypoxia, and toxic metabolites deposited in the environment^[^^50^^]^. These conditions, together with the expression of PD-L1 in tumor cells, impede T-cell metabolism and instead rise tumor metabolic activity^[^^51^^]^. There are also subsets of suppressive cells in the tumor microenvironment including MDSCs, which can also halt the antitumor response of T cells by reducing key amino acids such as arginine and tryptophan^[^^52^^]^. Moreover, different types of myeloid cells such as MDSCs, DCs, and tumor-associated macrophages release IDO into the tumor microenvironment; this enzyme converts tryptophan to its metabolites, especially kynurenine, and prevents the anti-tumor activity of T cells^[^^53^^]^. In addition, IDO can increase the expression of CFH and FHL-1^[^^54^^,^^55^^]^. FHL-1 levels is related to the increased recruitment of MDSCs and Treg cells, both of which cause immuno-suppression^[^^54^^]^. The hypoxic condition has been shown to induce the expression of CD39 and CD73 enzymes in various cell types in the tumor microenvironment. These ectonucleotidases break down the ATP molecules into adenosine, a ligand for purine receptors A2A and A2B that express on a diverse range of immune cells and enable the cells to suppress the antitumor immunity in T cells^[^^56^^,^^57^^]^. The Treg cells in most cancer tissues display a high expression level of CD36 in comparison to other tissues^[^^58^^]^. Also, a study on a mouse model with a genetic knockout of CD36 has shown as decrease in the infiltration of Treg cells into the tumor site, but the levels of infiltrated antitumor T cells increased^[^^58^^]^. Therefore, concentrated CD36 in Treg cells probably inhibits the metabolic fitness of Tregs in the tumor microenvironment and improves tumor prognosis^[^^59^^]^. In vitro studies have revealed that the increased levels of extracellular lactate and H⁺ in the tumor microenvironment can hamper proliferation, survival, cytotoxicity, and cytokine production in mouse and human CD8^+^ T cells, the fundamental players in eradicating tumor cells through triggering the signals from V-domain immunoglobulin suppressor of T-cell activation, an acidic pH-selective ligand for P-selectin glycoprotein ligand-1^[^^60^^]^. Another mechanism reported for tumor cells to interfere with T-cell activity is releasing oncometabolite, (R)-2-hydroxyglutarate, which disrupts T-cell receptor signaling, nuclear factor of the activated T cells, and polyamine biosynthesis in CD4^+^ and CD8^+^ T cells. This metabolite also increases methylation and modifies transcription profile by inhibiting dioxygenase enzymes such as histone demethylase^[^^61^^]^. A high rate of tumor cell necrosis causes an increment in potassium levels in the tumor microenvironment, which reduces the cytoplasmic levels of acetyl-CoA in T cells and limits T-cell function^[^^62^^]^. Most tumor cells express the enzyme iNOS, which causes the elimination of arginine within the tumor microenvironment^[^^63^^]^. In addition, M2 macrophages can also hydrolyze arginine by arginase expression and eventually cause arginine depletion^[^^64^^]^. Arginine plays a critical role in the function of T and natural killer cells, and decreasing arginine levels in TIME suppresses the function of effector killer T cells^[^^65^^]^. Mitochondrial metabolism is also crucial for T cells. Anoxia within the tumor microenvironment promotes mitochondrial fragmentation, decreases ATP manufacturing, and induces exhausted T cells^[^^66^^]^. High mitochondrial mass shows advanced respiratory capacity necessary to produce long-lived memory CD8^+^ T cells^[^^43^^]^. Consistent with this phenomenon, the ability of T effectors to generate memory T cells is impaired when the mitochondrial membrane fusion protein optical atrophy 1 is deleted in mice^[^^67^^]^. Consequently, mitochondrial fusion can enhance the survival of CD8^+^ T cells and convert the surface markers and bioenergic profile of these cells to memory T cells^[^^67^^]^. Current evidence or have confirmed that tumor cells use nanotubes to hijack the mitochondria of immune cells^[^^68^^]^, which results in the immune cell suppression, but the empowerment of tumor cells.

**Targeting T cell metabolism for cancer treatment**

**Fig. 3:**
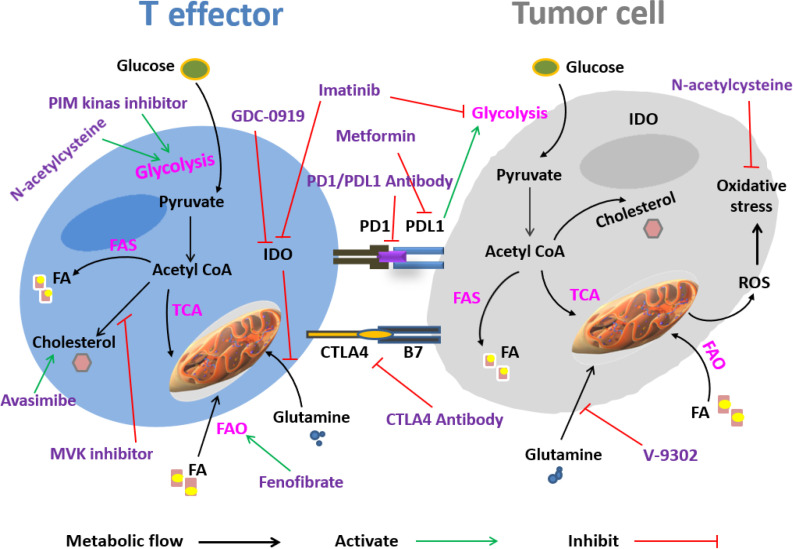
Therapeutic approaches for targeting T cell and tumor metabolism. Tumor cells compete with T cells for the uptake of essential nutrients including glucose, amino acids, and fatty acids, leading to food deprivation, hypoxia, and the production of toxic metabolites in the tumor microenvironment to further inhibit T cell function. Some drugs targeting the metabolic processes of T cells and tumor cells contribute to the antitumor effect, such as PD-1/PD-L1 antibodies, CTLA4 antibodies, metformin, fenofibrate, V-9302, Avasimibe, MVK inhibitor, GDC-0919, and imatinib. PD1, death protein 1; FA, fatty acid; ROS, reactive oxygen species; CTLA-4, cytotoxic lymphocyte antigen 4

Regarding the key role of T cells in defense against tumor cells, recent studies have focused on targeting metabolic pathways in order to fortify T-cell function and longevity. It is clear that the modulation of mTOR signaling in the tumor microenvironment has significant effects on T-cell metabolism and function; thus, the accurate understanding of how targeting tumor cells alter T-cell immunity may be helpful^[^^69^^]^. Research has also examined the effects of other molecular targets on metabolic pathways involving in T cell activation^[^^70^^,^^71^^]^. Factor 4-1BB is a co-stimulatory molecule detectable in most immune cells and is highly inducible in DCs and Treg cells^[^^72^^]^. Interaction of 4-1BB with its ligand induces strong NF-κB activation to enhance CTL function^[^^73^^]^. This molecule also increases fatty acid and glucose consumption and boosts mitochondrial biogenesis. In fact, 4-1BB supports antitumor immunity by enhancing T-cell mitochondrial function^[^^74^^,^^75^^]^. Immunotherapy with 4-1BB agonist antibodies has long been used in several clinical trials^[^^76^^]^. As mentioned earlier, high lactate levels and low pH are associated with a decrease in the effectiveness of immunotherapy. Hence, inhibiting lactate production may raise immunotherapy effectiveness by restoring T-cell function while repressing Treg activity^[^^77^^,^^78^^]^. The gut microbiota can turn non-absorbable polysaccharide into short-chain fatty acids, such as acetate, butyrate, and propionate^[^^79^^]^. He et al.^[^^80^^]^ confirmed that butyrate, the microbial metabolite of the intestine, stimulates the IL-12 signaling pathway to promote CD8^+^ cell-mediated antitumor response, leading to the improved efficacy of oxaliplatin treatment. Accordingly, the oxaliplatin-sensitive sufferers display higher ranges of serum butyrate in comparison to sufferers with tumors resistant to oxaliplatin. Altogether, exploring new strategies to inhibit tumor cell metabolism and improve the ability of T cells to obtain nutrients might be a challenge for future studies (Table 1 and Fig. 3). 

***Targeting glucose metabolism in T cells***

Expression of the programmed cell death, PD-L1 on tumor cells activates the Akt/mTOR pathway to boost glycolytic metabolism in tumor cells. In contrast, the interaction between PD-L1 and its receptor PD-1 on T cells makes these cells unable to utilize glucose and branched-chain amino acids, but instead, the levels of FAO increase. Monoclonal antibodies that block the PD-1/PD-L1 checkpoint may restore glucose levels in the tumor microenvironment, leading to an increase in T-cell glycolysis and IFN-γ production^[^^30^^,^^81^^]^. Therefore, inhibitors of PD-1/PD-L1 interaction can help T cells eliminate tumor cells by modulating their metabolism^[^^81^^,^^82^^]^. Similar to PD-1, LAG-3 is another inhibitory molecule on T cells. Previte et al.^[^^83^^]^ reported that naive T cells with LAG-3 deficiency showed increased oxidation and glycolytic metabolism due to an increase in the mitochondrial mass. Therefore, targeting LAG-3 may provide a new strategy for antitumor therapy. Cytotoxic T lymphocyte antigen 4 is expressed on the surface of chronically activated T cells. This inhibitory molecule suppresses the PI3K/Akt/mTOR signaling pathway and prevents glucose uptake in T cells^[^^84^^,^^85^^]^. Imatinib, a BCR-ABL kinase inhibitor, has been indicated to have different effects on T-cell metabolism. According to Beckermann et al.^[^^86^^]^, this medication activates CD8^+^ T cells, while induces Treg cell apoptosis by reducing IDO expression. Moreover, metformin as a classic regulator of glucose metabolism, has a direct anti-tumor activity and an indirect effect on strengthening CTL function. This drug blocks the mTOR pathway and interferes with tumor glycolysis and thus tumor growth^[^^87^^]^. Also, metformin downregulates the PD-L1 expression on the tumor cells and increases the cytotoxic function of CTL^[^^87^^]^. The proviral integration site for moloney murine leukemia virus PIM is a serine/threonine kinase involved in regulating T-cell glucose metabolism. Inhibiting the PIM kinase, increases mTORC1 activity and leads to higher uptake of glucose by T cells and enhances the antitumor immunity^[^^88^^]^.

***Targeting lipid metabolism in T cells***

Some studies have noted the role of lipid metabolism, including intracellular cholesterol levels, in regulating tumor cell as well as T-cell activity. The effect of some lipid-metabolizing drugs, such as statins, on T cells is still controversial. According to the literature, statins can negatively affect tumor cells by inhibiting lipid metabolism and also affect T cells by reducing the cholesterol levels^[^^89^^]^. One of the metabolic pathways for cholesterol synthesis is the MVK pathway. Some studies have reported that tumor cells using this pathway can activate the immune responses, introducing a new antitumor target for immunotherapy. MVK is also important for T-cell activation in an Akt/mTOR signaling-dependent manner^[^^90^^]^. Avasimibe, as an acyl-CoA acyltransferase inhibitor, prevents cholesterol esterification and enhances the level of intracellular free cholesterol tumor cells, thus inhibiting the proliferation and metastasis in tumor cells and increasing the activity of CD8^+^ T cells. Avasimibe has also been used to treat cancer in murine models of tumors and shown considerable antitumor effects. A combination of avasimibe with PD-1 antibodies has shown a better efficacy in regressing tumor progression rather than monotherapy^[^^30^^,^^91^^]^. Fenofibrate is an activator of peroxisome proliferator-activated receptor alpha, involved in regulating lipid metabolism^[^^92^^]^, thereby increasing FAO in T cells and reversing the suppression of T cells in the tumor microenvironment^[^^93^^]^. Liu et al.^[^^94^^]^ discovered that both Treg cells and tumor cells in the tumor microenvironment could modify the lipid metabolism in T cells by increasing the expression of group IVA phospholipase A, a change that eventually results in T cell senescence. Senescent T cells have little capacity to kill tumor cells. Inhibiting group IVA phospholipase A in T cells from breast cancer and melanoma models showed that the lipid metabolism reprogrammed in T cells and the antitumor ability increased^[^^94^^]^. Therefore, targeting lipid metabolism in T cells might synergistically enhance the therapeutic impact of most cancer treatments.

**Table 1:** Therapeutic approaches for targeting T cell and tumor metabolism

**Treat**	**Target**	**Signaling pathway**	**Effect**
PD-1/PD-L1 antibodies	PD-1/PD-L1	PI3K/Akt/mTOR	Teffs: increase FAO Tumor: inhibit glycolysis
			
CTLA-4 antibodies	CTLA-4	PI3K/Akt/mTOR	Teffs: inhibit glucose uptake
			
Imatinib	BCR-ABL kinase/IDO	BCR/ABL IDO	Teffs: activation Treg: apoptosis Tumor: switch from glycolysis to OXPHOS
			
CTLA-4 antibodies	CTLA-4	PI3K/Akt/mTOR	Teffs: inhibit glucose uptake
			
Imatinib	BCR-ABL kinase/IDO	BCR/ABL IDO	Teffs: activation Treg: apoptosis Tumor: switch from glycolysis to OXPHOS
			
Metformin	PD-L1	LKB1-AMPK system mTOR	Tumor: down-regulate PD-L1 expression
			
PIM kinase inhibitor	PIM kinase	mTORC1	Teffs: increase glucose uptake
			
MVK inhibitor	MVK	PI3K/Akt/mTOR	Teffs: activation Tumor: inhibition
			
Avasimibe	ACAT-1	Cholesterol esterification	Teffs: activation Tumor: inhibit the proliferation and Metastasis
			
GDC-0919	IDO1	Tryptophan	Teffs: relieve CD8^+^ T-cell inhibition
			
INCB024360	IDO	Tryptophan	Teffs: increase proliferation and IFN-γ production
			
N-acetylcysteine	FOXO1	PI3K/Akt/mTOR	Teffs: affect granzyme B secretion and PD-1 expression
			
V-9302	Glutamine transporter	Glutamine	Teffs: no affect Tumor: inhibit the proliferation

Teff, effector T cell

***Targeting T cell amino acid metabolism***

IDO1 catalyzes oxidation of tryptophan to kynurenine and is partly responsible for the acquired immune tolerance to cancer. A research performed on patients with colon cancer highlighted a reverse association between IDO expression and T-cell infiltration into the tumor microenvironment. This study also observed a lower rate of survival in the patients with higher IDO levels^[^^95^^]^. Navoximod (GDC0919) is a new IDO inhibitor tested in several tumor models with potential immunomodulatory properties. This drug can improve CD8^+^ T cell cytotoxicity by reducing the amount of kynurenine^[^^96^^]^. In addition, the combination treatment of Navoximod with chemotherapy, radiotherapy, or vaccine leads to the progressed antitumor response^[^^96^^]^. Another IDO inhibitor named INCB024360, results in the increased T-cell proliferation and IFN-γ production in mouse models^[^^97^^]^. Administration of IDO inhibitors can substantially increase the production of cytokines including IL-2, TNF-α, and IFN-γ in CD8^+^ T cells and enhance their functions^[^^98^^]^. Therefore, using IDO inhibitors is one of the strategies currently under investigation for activating T cells in turmeric conditions. Studies have shown that PD-1 expression reduces when CD8^+^ T cells are cultivated under glutamine-restricted conditions^[^^99^^]^. Decreased expression of PD-1 results in boosting the activity of CD8^+^ T cells, suggesting a helpful strategy to improve immunotherapy^[^^100^^]^. Similarly, NAC can inhibit the expression of FOXO1 by activating the PI3K/Akt signaling pathway in CD8^+^ T cells and increase secretion of granzyme-B and, consequently, the anti-turmeric capacity of these cells^[^^101^^]^. NAC is an analogue of cysteine, which is required for glutathione synthesis. Glutathione possesses antioxidant properties and counteracts the oxidative stress observed in the tumor microenvironment^[^^102^^]^. Glutamine is also a target metabolite in cancer therapy. In a study on mouse TNBC model, T effectors and tumor cells compete for glutamine from the microenvironment; deletion of glutaminase in tumor cells, an enzyme crucial for glutamine metabolism, activates T cells and enhances antitumor immune responses^[^^103^^]^. In addition, V-9302, an inhibitor of the glutamine transporter, can specifically inhibit glutamine uptake in TNBC cells, but not in antitumor T cells, representing a promising therapeutic strategy against TNBC^[^^103^^]^.

Metabolic changes in the tumor microenvironment can intensively affect T cells, from activation to their differentiation and function. Therefore, a better understanding of immunometabolic events is of utmost importance to achieve new anticancer therapeutics. Based on the preclinical and clinical studies, the longevity and stability of T cells used in immunotherapy is the main factor determining the effectiveness of therapy. Further studies are needed to develop techniques for manipulation of the metabolic pathways in order to enhance T cell responses against cancer progression. 

### Conclusion

To sum up, studies on immunometabolism in T lymphocytes can not only facilitate basic research but also provide potential targets in drug discovery against immune-related disorders, including cancer.

## DECLARATIONS

### Ethical statement

Not applicable. 

### Data availability

The raw data supporting the conclusions of this article are available from the authors upon reasonable request.

### Author contributions

JA: performed the literature search and data analysis and drafted the manuscript; FS: had the idea for the article and critically revised the work; MM: critically revised the work; HF: critically revised the work; MR: performed the literature search and data analysis. 

### Conflict of interest

 None declared.

### Funding/support

This review received no financial support.
